# A bioprocess optimization study to enhance the production of Menaquinone-7 using *Bacillus subtilis* MM26

**DOI:** 10.1186/s12934-025-02735-8

**Published:** 2025-05-16

**Authors:** Maneesha M, Subathra Devi C

**Affiliations:** Department of Biotechnology, School of Bio Sciences and Technology, Vellore Institute of Technology, Vellore, 632014 Tamil Nadu India

**Keywords:** Menaquinone-7, *Bacillus subtilis*, Optimization, RSM, Production, OFAT, Home-made wine

## Abstract

**Background:**

Menaquinone-7 (MK-7) has a vital significance in promoting human health and tackling several global health concerns which makes its production extremely important. MK-7 is not easily accessible at a reasonable cost due to the poor fermentation yields and the existence of several laborious downstream unit processes. Efficient manufacturing methods are essential to meet the global requirements due to the increasing demand in the pharmaceutical and nutraceutical industries. This research study focuses on the enhanced production of MK-7 from *Bacillus subtilis* MM26 isolated from fermented home-made wine.

**Results:**

A suitable MK-7 production medium for *Bacillus subtilis* MM26 was determined and the yield was found to be 67 ± 0.6 mg/L. The one factor at a time (OFAT) results showed that medium containing lactose, glycine, with a pH 7, a temperature of 37 °C, and an inoculum size of 2.5% (2 × 10⁶ CFU/mL) was optimal synthesis of MK-7. RSM indicated that incubation time, carbon and nitrogen sources were the factors significantly affecting the MK-7 yield. RSM predicted optimal conditions, which yielded a maximum concentration of 442 ± 2.08 mg/L of MK-7.

**Conclusions:**

The outcomes of this study demonstrated the potential of *Bacillus subtilis* MM26 in large-scale industrial production of MK-7. The yield of MK-7 was amplified efficiently by integration of OFAT and RSM, paving the way for cost-efficient industrial production.

## Background

Menaquinone-7 is a member of vitamin K2 that has gained interest over recent years due to its vital role in human health [1]. Vitamin K2 often referred as menaquinones are members of vitamin K family which is used by the body for blood clotting, to develop healthy bones and to diminish the risk of heart diseases and vascular calcification [2]. MK-7 is a subclass of menaquinones that set it apart from other variants such as MK-4 due to its seven isoprenoid side chain units [3]. The enhanced half-life and bioavailability of MK-7 compared to other menaquinones has led to recent growth in use and sales of MK-7 [4]. The natural sources of MK-7 include extracts of plants, foods or products derived from animals. High MK-7 content is observed in a traditional Japanese fermented soybean cuisine known as natto. It is also produced by various microbes, such as specific strains of *Bacillus* and lactic acid bacteria [5, 6]. Significantly, *Bacillus subtilis* is capable of producing MK-7 industrially [7].

MK-7 activates proteins that are reliant on vitamin K, including osteocalcin and matrix Gla-protein (MGP) [8]. These proteins are essential for calcium metabolism and to prevent its deposition on the lining of blood vessel [9]. MK-7 functions as an important energy producing component in bacteria as it is acting as a carrier of electron within the electron transport chain [10]. Studies show that in humans, MK-7 supplementation can improve arterial stiffness [11] and regulates bone metabolism for key prevention of osteoporosis [12]. Furthermore MK-7 contributes to cardiovascular health by inhibiting vascular calcification, type 2 diabetes, rheumatoid arthritis, chronic kidney disease, depression in women with PCOS, and Alzheimer’s disease [1, 9]. Since Mk-7 is a vital nutrient, the development and industrial production of MK-7 have become a key area of research [13].

MK-7 can be synthesized through chemical synthesis as well as microbial fermentation. Although chemical methods are generally more cost-effective, natural synthesis is favored. Fermentation-based production, which utilizes microorganisms for industrial synthesis of MK-7, promotes the sustainability of the environmental. The problems associated with this are low yield of vitamin and complex downstream processing, leading to higher production cost and making it less affordable because of the elevated price of the product [14, 15]. Numerous wild-type microorganisms were isolated from distinct environments and were subjected to their industrial production of MK-7. Most of these native vitamin-producing strains are generally low-yielding and produce vitamin poorly or are genetically unstable. Maximum vitamin production and stability can be achieved through metabolic engineering of the microorganisms. Bioprocess engineering approaches include optimization of fermentation conditions and media composition to enhance product yield [6] *Bacillus subtilis* MM26, isolated from fermented homemade wine, was used in the current study for the MK-7 synthesis. The key production medium components, including pH, temperature, inoculum size, carbon and nitrogen sources were optimized to maximize the MK-7 yield. Response surface methodology was utilized to improve the production process. The aim of this study was to enhance MK-7 synthesis and develop an ideal medium to achieve its overproduction by *Bacillus subtilis* MM26.

## Materials and methods

### Microorganisms and seed culture Preparation

The *Bacillus subtilis* MM26 culture (accession number: PP268148) isolated from fermented home-made wine was preserved in nutrient agar slants at 4ºC, and sub-cultured every 30 days. A spore suspension of *Bacillus subtilis* MM26 was prepared and inoculated into a conical flask containing 100 mL nutrient broth liquid medium, with the pH 7. The culture was incubated for 24 h at 37 °C at 120 rpm.

### Production of MK-7

The Luria Bertani (LB) broth [16], nutrient broth, Tryptic soy broth (TSB) [17] and the production medium [6] composed of 0.06 g of K_2_HPO_4_, 1.89 g of soy peptone, 0.5 g of yeast extract and 0.5 mL of glycerol were used for the MK-7 production (100 mL). 100 µL of *Bacillus subtilis* MM26 was inoculated into all the four media and the media was kept for incubation at 37 °C for 5 days at 120 rpm. The growth of *Bacillus subtilis* MM26 was observed by determining the OD at 600 nm with a UV spectrophotometer, and MK-7 levels were subsequently quantified by performing HPLC [18].

### Extraction of MK-7

20 mL of culture broth was used for each extraction. The culture broth was sonicated for 4 min with a pulse mode of 10s on and 5s off and the broth was centrifuged for 10 min at 6000 rpm. To the 20mL supernatant, 26.6 mL of n-hexane and 13.4 mL of isopropanol was added as extraction solvents and kept in shaking incubator for 1 h at 37 °C. The mixture was centrifuged for 10 min at 6000 rpm. The solution was evaporated using rotary evaporator [19].

### MK-7 analysis

#### HPLC and FT-IR

The presence of MK-7 was ascertained by employing high performance liquid chromatography. The mobile phase used in the HPLC was methanol and acetonitrile (1:1) with a retention time of 4.9 min at 254 nm and compared to the standard MK-7 [18]. FTIR spectra of MK-7 was measured using a Perkin Elmer FTIR spectrometer. The spectra were compared with the standard MK-7 [20].

#### Optimization

To maximize the MK-7 production in *Bacillus subtilis* MM26, five different factors such as pH, inoculum size, temperature, carbon and nitrogen sources were assessed to optimize the production medium. Glycerol, initially used as the carbon source, was replaced with alternatives such as fructose, dextrose, lactose and maltose. Similarly in order to optimize nitrogen source, soy peptone was substituted with beef extract, tryptone, peptone, and glycine. The temperature range for optimization was set between 25 °C and 40 °C, while pH values from 6 to 8 were tested to evaluate the optimal pH for MK-7 synthesis. Inoculum sizes ranging from 0.5 to 2.5% were also evaluated. The primary parameters for optimization were the MK-7 synthesis and the growth rate of *Bacillus subtilis* MM26 [21, 22].

### Statistical optimization of MK-7 using response surface methodology

The Box–Behnken statistical approach was employed to analyze the impacts of incubation time, carbon, and nitrogen source on MK-7 production. Both the independent and combined effects of these elements were assessed using this methodology. Design-Expert 13 software (Stat-Ease Inc., Minneapolis, MN, USA) was employed to develop the experimental design. Every variable had three levels: lactose (g/L) at 3, 6, and 9; glycine (g/L) at 12, 17.5, and 23; and incubation time (h) at 60, 120, and 180. 17 experimental runs were conducted in which each medium was made in 100 mL. 2.5% inoculum of fresh bacterial culture was added, followed by incubation at 37 °C at 120 rpm. Each experiment was carried out in triplicate, and the mean values were utilized for response surface methodology (RSM). The experimental and expected responses were compared using analysis of variance (ANOVA). A second-order polynomial equation was utilized to interpret the data in relation to the responses and thus, this was followed by multiple regression analysis for the fitting. In order to validate the findings, a duplicate experiment was conducted under the optimal conditions determined through response surface optimization and the predicted and observed responses were compared [23, 24].

## Results

### MK-7 production

*Bacillus subtilis* MM26 yielded MK-7 of 67 ± 0.6 mg/L in the medium comprises of 5% glycerol, 18.9% soy peptone, 0.06% K_2_HPO_4_, 5% yeast extract. The MK-7 production was determined to be 64 ± 1.15 mg/L on TSB. On NB medium, MK-7 production was observed to be 60 ± 1.45 mg/L and on LB medium the strain produced MK-7 of 52 ± 0.76 mg/L. The trials’ average was determined after the experiments were conducted in triplicate. The final evaluation employed the error bars and the standard deviation (SD) that had been determined.

### Analytical assays

#### HPLC

**Fig. 1:**
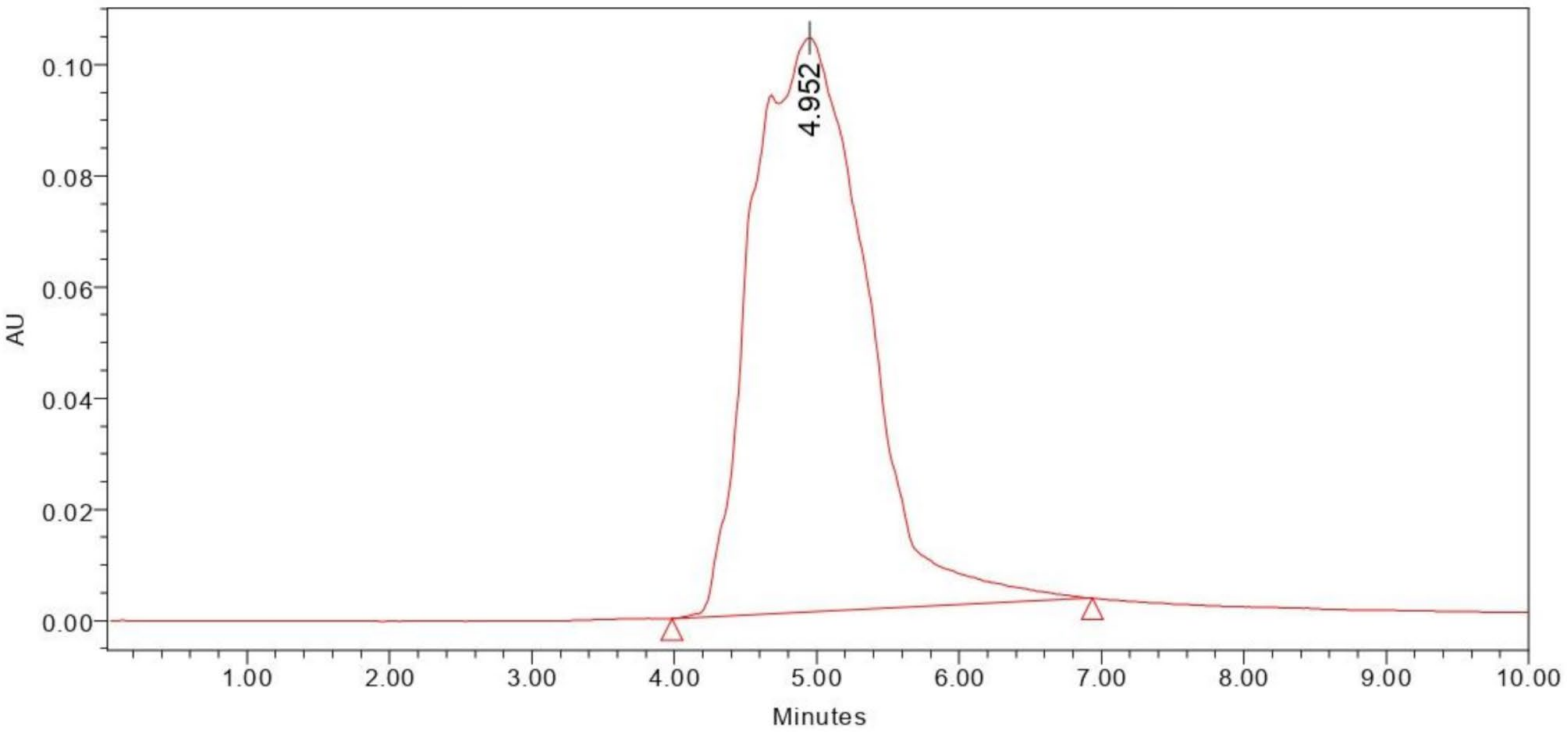
HPLC chromatogram of menaquinone 7 isolated from *Bacillus subtilis* MM26

The vitamin, MK-7, yielded by *Bacillus subtilis* MM26, was determined using HPLC. The test sample showed the retention time of 4.9 min same as the standard MK-7. The sample was validated as MK-7 since both the RT values were similar (Fig. 1).

#### FT-IR

**Fig. 2:**
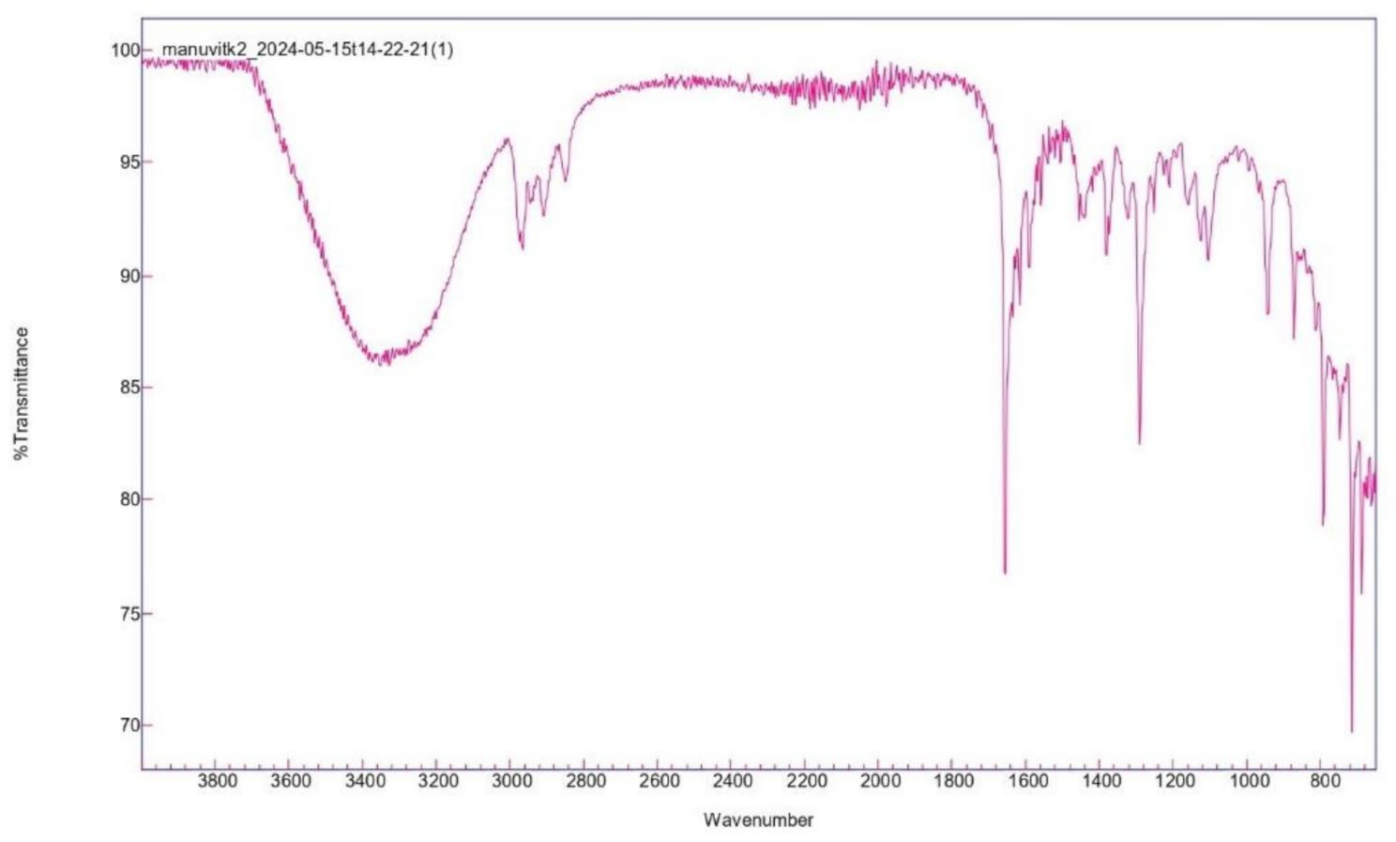
FT-IR spectra of menaquinone 7 isolated from *Bacillus subtilis* MM26

A distinctive carbonyl peak was seen at 1660 cm^− 1^ in the standard MK-7 FTIR spectra. The value 1660 cm − 1 of menaquinone-7 represents the carbonyl group on the naphthoquinone ring, whereas the amide II group is represented by a value of 1536 cm^− 1^. The MK-7 produced by *Bacillus subtilis* MM26 showed the spectra same as the standard MK-7 (Fig. 2).

### Optimization

#### Carbon sources

**Fig. 3:**
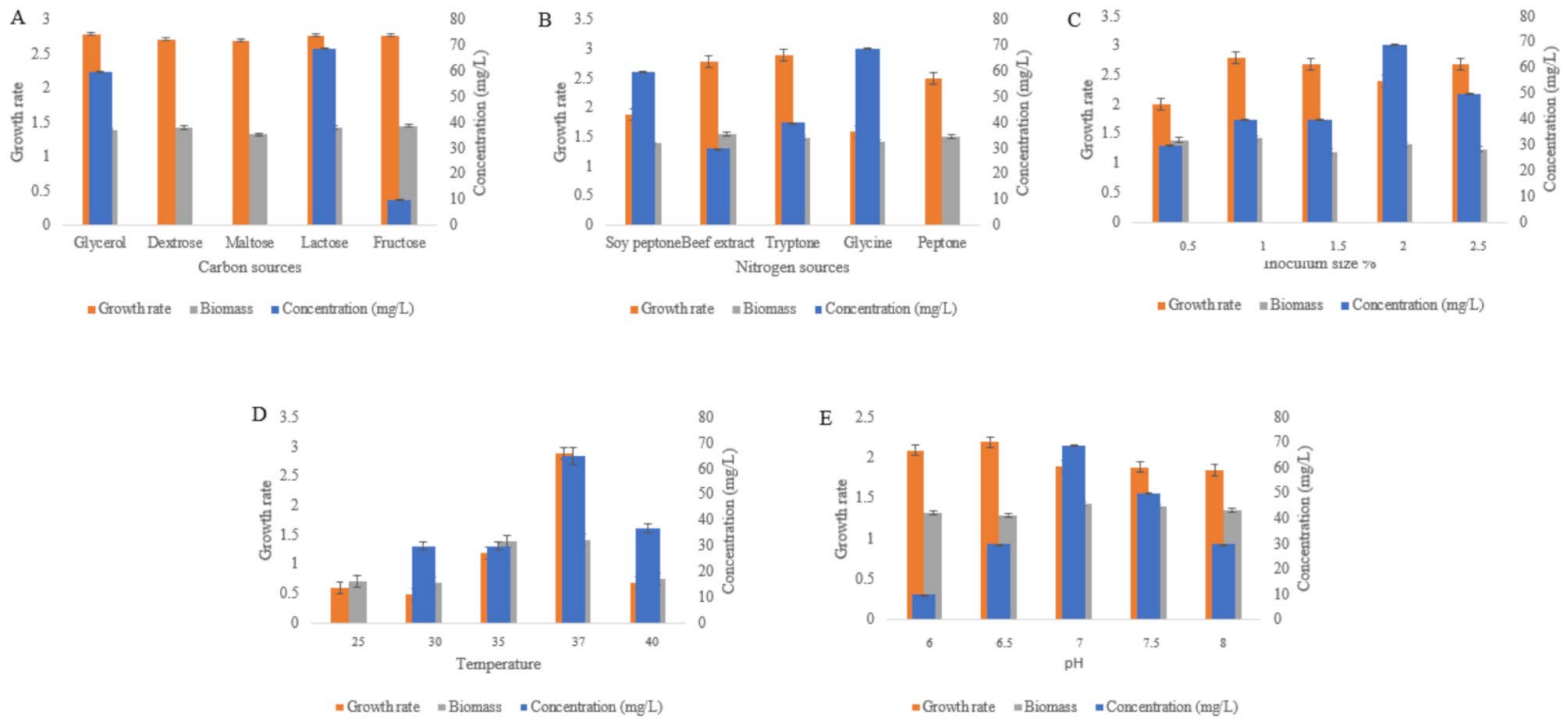
Optimization of menaquinone 7 using various parameters (**A**) Carbon sources, (**B**) Nitrogen sources, (**C**) Inoculum size, (**D**) Temperature, (**E**) pH

Based on the data, lactose has been proven to be the most ideal carbon source. When lactose was incorporated to the medium, the strain *Bacillus subtilis* MM26 generated MK-7 of 69 ± 1.20 mg/L. The second-best carbon source was found to be glycerol, and the strain produced Mk-7 of 60 ± 2.08 mg/L. The strain produced 10 ± 0.7 mg/L of MK-7 when fructose was used as the source of carbon. However, when dextrose and maltose were introduced as carbon sources, the strain was not able to produce MK-7 (Fig. 3A).

#### Nitrogen sources

Glycine has been shown to be the most appropriate source of nitrogen based on the data. When added to the medium, the strain *Bacillus subtilis* MM26 produced 69 ± 1.79 mg/L of MK-7. Soy peptone was determined to be the second-best source of nitrogen, and the strain yielded Mk-7 of 60 ± 2.00 mg/L. The strain produced 40 ± 1.85 mg/L and 30 ± 1.45 mg/L of MK-7 when tryptone and beef extract were used as the source of nitrogen. But when peptone was added as a source of nitrogen, the strain was not able to produce MK-7 (Fig. 3B).

#### Inoculum size

The concentration of MK-7 rose concurrently with an increase in the proportion of inoculum. The optimal inoculum size was found to be 2.5%, and a content of MK-7 of 68 ± 0.57 mg/L was produced. Inoculating the medium with 0.5% and 1% of inoculum, the MK-7 production was decreased to 30 ± 1.73 mg/L and 40 ± 0.88 mg/L, respectively. The strain produced MK-7 of 40 ± 0.33 mg/L and 50 ± 1.20 mg/L, when the proportion of inoculum were 1.5% and 2% (Fig. 3C).

#### Temperature

The medium containing the *Bacillus subtilis* MM26 was incubated at various temperatures in order to investigate the impact of temperature. The ideal temperature for MK-7 synthesis was determined to be 37 °C. Under 37 °C, there was a greater increase in *Bacillus subtilis* MM26 growth and MK-7 synthesis (65 ± 2.76 mg/L). When incubated at 30 °C and 35 °C, the strain yielded MK-7 of 30 ± 0.5 mg/L and 30 ± 1.45 mg/L, respectively. The quantity of MK-7 detected in a medium incubated at 40 °C was 37 ± 1.2 mg/L. At 25 °C, the strain was unable to produce MK-7 (Fig. 3D).

#### pH

In order to ascertain the impact of pH, *Bacillus subtilis *MM26 was inoculated into media with varying pH values. Based on the results, the strain produced maximum MK-7 of 69 ± 0.57 mg/L at neutral pH 7. The strain produced a content of MK-7 of 50 ± 0.33 mg/L in a pH 7.5 medium. Lesser concentration of MK-7 such as 30 ± 1.2 mg/L and 30 ± 0.66 mg/L were obtained from pH 6.5 and pH 8 medium, respectively. At pH 6, the strain produced 10 ± 0.88 mg/L of Mk-7 (Fig. 3E).

### Statistical optimization of MK-7 by response surface methodology

To maximize the MK-7 production, the most fitting composition was determined using Box-Behnken statistical analysis. A total of 17 runs were conducted using incubation time as one factor and varying amounts of lactose and glycine as other two factors (Table 1). The maximum MK-7 yield (442 ± 2.08 mg/L) was achieved in the 12th run. Statistical analysis indicated that the quadratic model was the best fit for the data, demonstrated by its superior F value of 27.76. A second-order polynomial equation is as follows:

MK-7 concentration (mg/mL) = + 0.1440–0.0181*A -0.1295*B + 0.0309*C + 0.0230*A*B -0.0178*A*C -0.0530*B*C -0.0724*A^2^ + 0.0749*B^2^ -0.0109*C^2^ where lactose, glycine, and incubation time are the three test variables, and their corresponding coded words are A, B, and C.

**Table 1:** MK-7 concentration in each run

Std	Run	Factor 1 A: Lactose (g/L)	Factor 2 B: Glycine (g/L)	Factor 3 C: Incubation (H)	Response 1 MK-7 (mg/L)
1	1	3	12	120	315 ± 3.78
9	2	6	12	60	240 ± 2.88
6	3	9	17.5	60	048 ± 0.77
7	4	3	17.5	180	109 ± 1.54
10	5	6	23	60	080 ± 0.56
17	6	6	17.5	120	130 ± 2.00
5	7	3	17.5	60	046 ± 0.83
15	8	6	17.5	120	180 ± 3.17
4	9	9	23	120	024 ± 0.21
3	10	3	23	120	017 ± 1.24
13	11	6	17.5	120	169 ± 0.60
11	12	6	12	180	442 ± 2.08
14	13	6	17.5	120	122 ± 0.87
16	14	6	17.5	120	119 ± 1.00
12	15	6	23	180	070 ± 1.96
2	16	9	12	120	230 ± 2.60
8	17	9	17.5	180	040 ± 0.94

According to ANOVA, the F test had the highest significance with a low probability of 0.0001, showing that the likelihood of a “model F-value” of this size resulting due to noise was only 0.01%. The (A, B, and C) variables in the model were determined to be pivotal according to the *p* values obtained from multiple regression analysis (Table 2). The analyzed model showed that the F value of variable B is 165.19, which had a higher influence on the increased MK-7 synthesis than A and C. Comparing the F value of variable A (3.24) to the F value of variable C (9.39), the former had the least impact on the production of MK-7. The “Lack of Fit” F-value is 1.02, suggesting that the pure error has no impact on the lack of fit. The model is considered appropriate because the lack of fit is not statistically significant. The fit statistic’s R2 value is 0.9727. The values of adjusted R^2^ and the predicted R^2^ is 0.9377 and 0.7872. The variation between the adjusted and predicted R^2^ value is < 0.2, indicating that the result is suitable for study. The ratio of signal-to-noise is computed using “Adeq Precision.” Consequently, the ratio of 18.7235 showed that the signal is suitable and that the design space may be explored with this model.

**Table 2:** ANOVA table for the quadratic model

Source	Sum of Squares	Df	Mean Square	F-value	*p*-value	
Model	0.2029	9	0.0225	27.76	0.0001	Significant
A-Lactose	0.0026	1	0.0026	3.24	0.1151	
B-Glycine	0.1342	1	0.1342	165.19	< 0.0001	
C-Incubation period	0.0076	1	0.0076	9.39	0.0182	
AB	0.0021	1	0.0021	2.61	0.1505	
AC	0.0013	1	0.0013	1.55	0.2530	
BC	0.0112	1	0.0112	13.83	0.0075	
A²	0.0221	1	0.0221	27.16	0.0012	
B²	0.0236	1	0.0236	29.06	0.0010	
C²	0.0005	1	0.0005	0.6131	0.4593	
Residual	0.0057	7	0.0008			
Lack of Fit	0.0025	3	0.0008	1.02	0.4734	not significant
Pure Error	0.0032	4	0.0008			
Cor Total	0.2086	16				

**Fig. 4:**
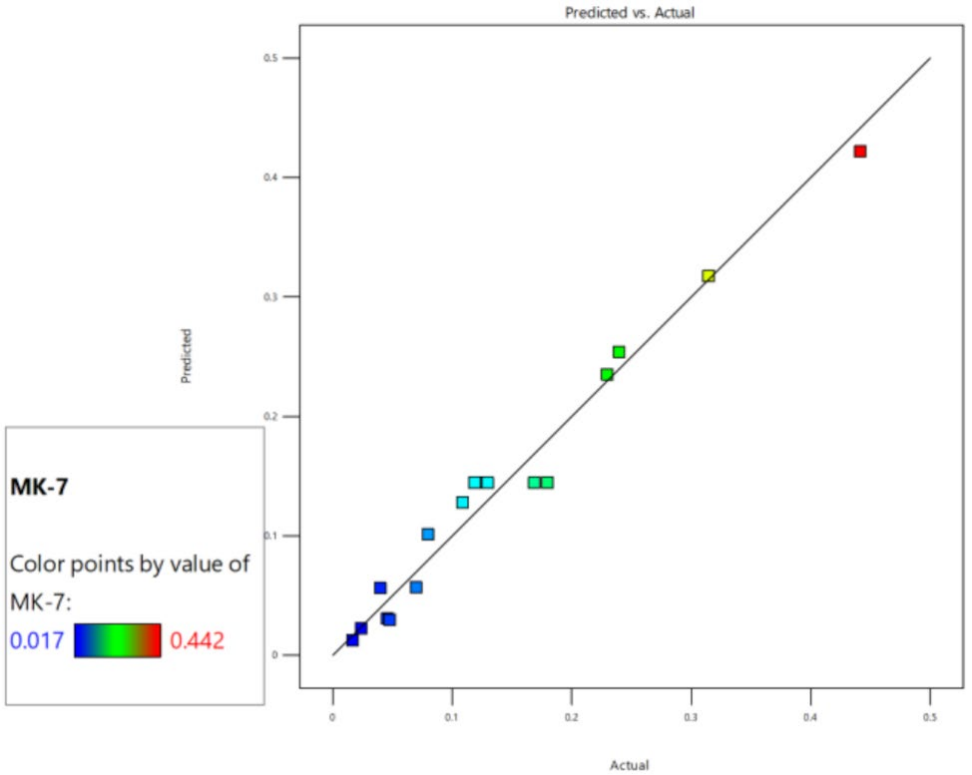
Predicted vs. actual graph

The predicted and actual MK-7 production outcomes were aligned with each other demonstrating the high precision and reliability of the study. When the experimental data was compared to the expected values of MK-7 production, which were computed using ANOVA, it was shown that the predicted and actual response values were highly similar (Fig. 4). Response surface plots (Fig. 5A, 5B and 5C) were generated using the equation given in the model in order to examine the correlations between the variables and ascertain the ideal concentration of each component for maximal MK-7 synthesis by *Bacillus subtilis*. The substantial effects of several factors, either alone or in combination, on the production of MK-7 is illustrated in 3D graph. The interactions of lactose (3–6 g/L) and glycine (12–23 g/L) with an incubation time of 180 h are shown in Fig. 5A. The interactions of lactose and incubation time by keeping a steady glycine concentration is shown in Fig. 5B. Figure 5C depicts the interactions between glycine and incubation time with a steady concentration of lactose. The findings showed that lactose, glycine and incubation time, all had a major impact on the synthesis of MK-7 and were essential in regulating MK-7 biosynthesis.

**Fig. 5A:**
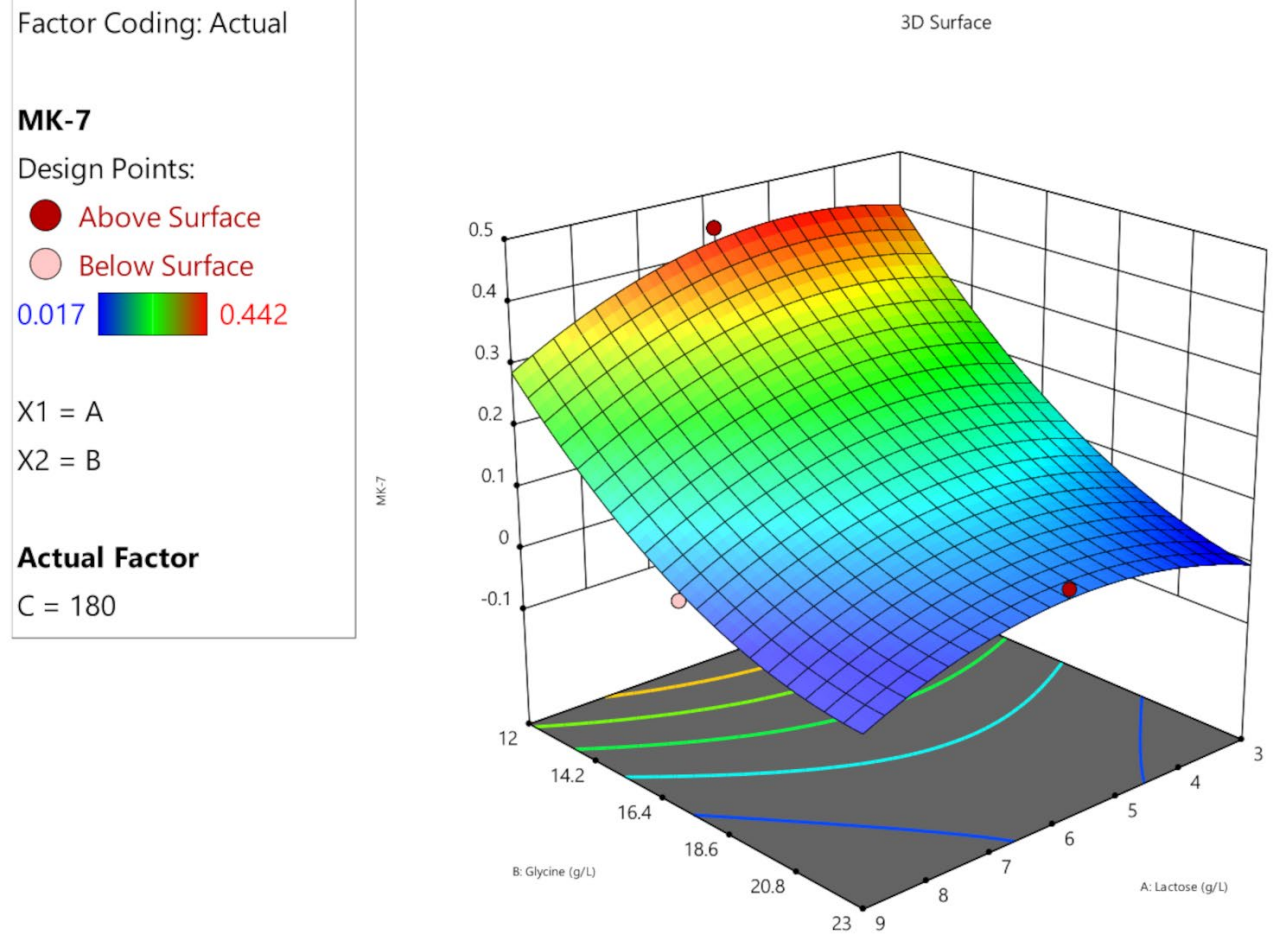
3-D response surface plot showing the interaction between carbon and nitrogen source for the optimization of MK-7 production medium

**Fig. 5B:**
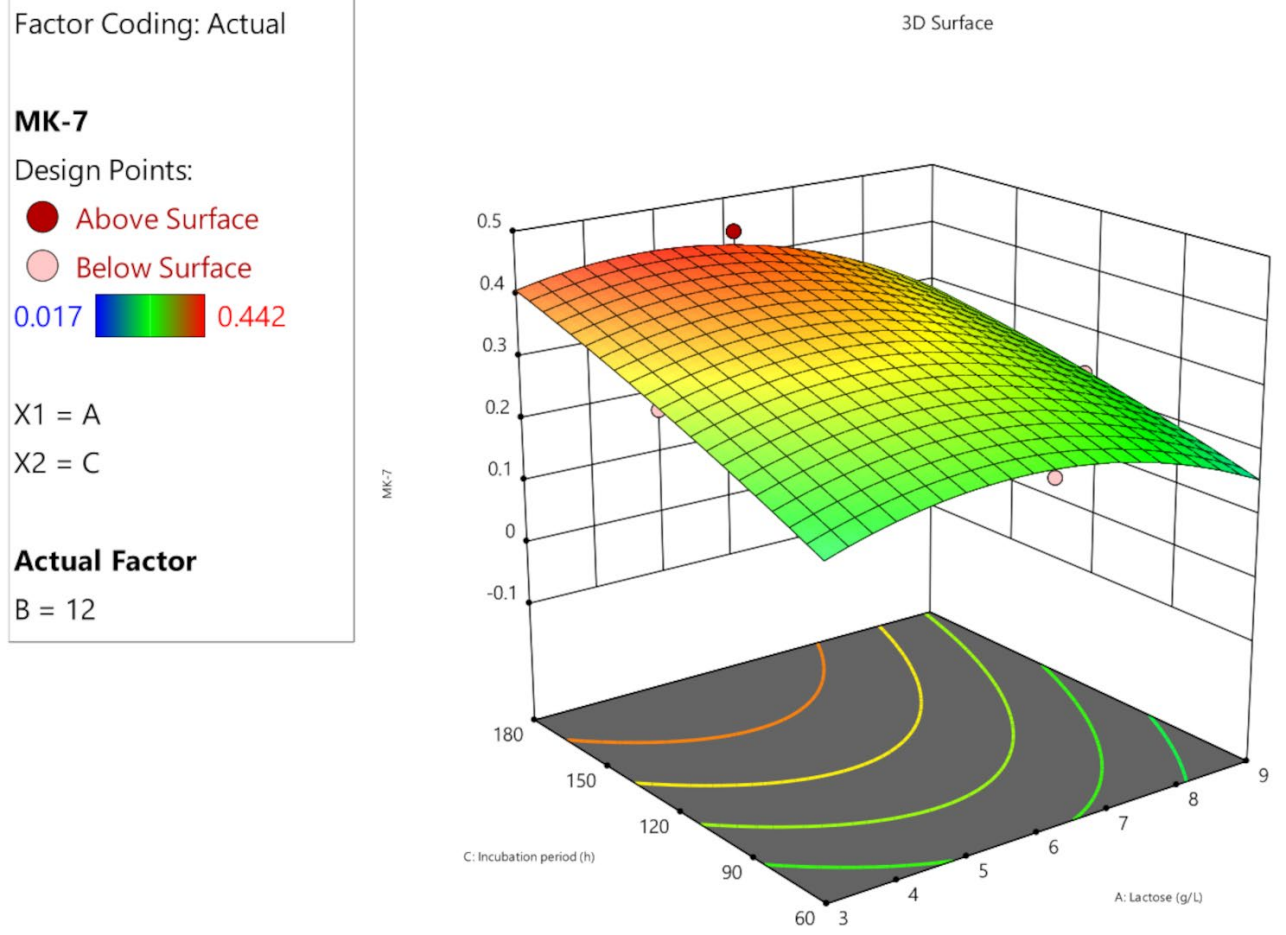
3-D response surface plot showing the interaction between carbon source and incubation time for the optimization of MK-7 production medium

**Fig. 5C:**
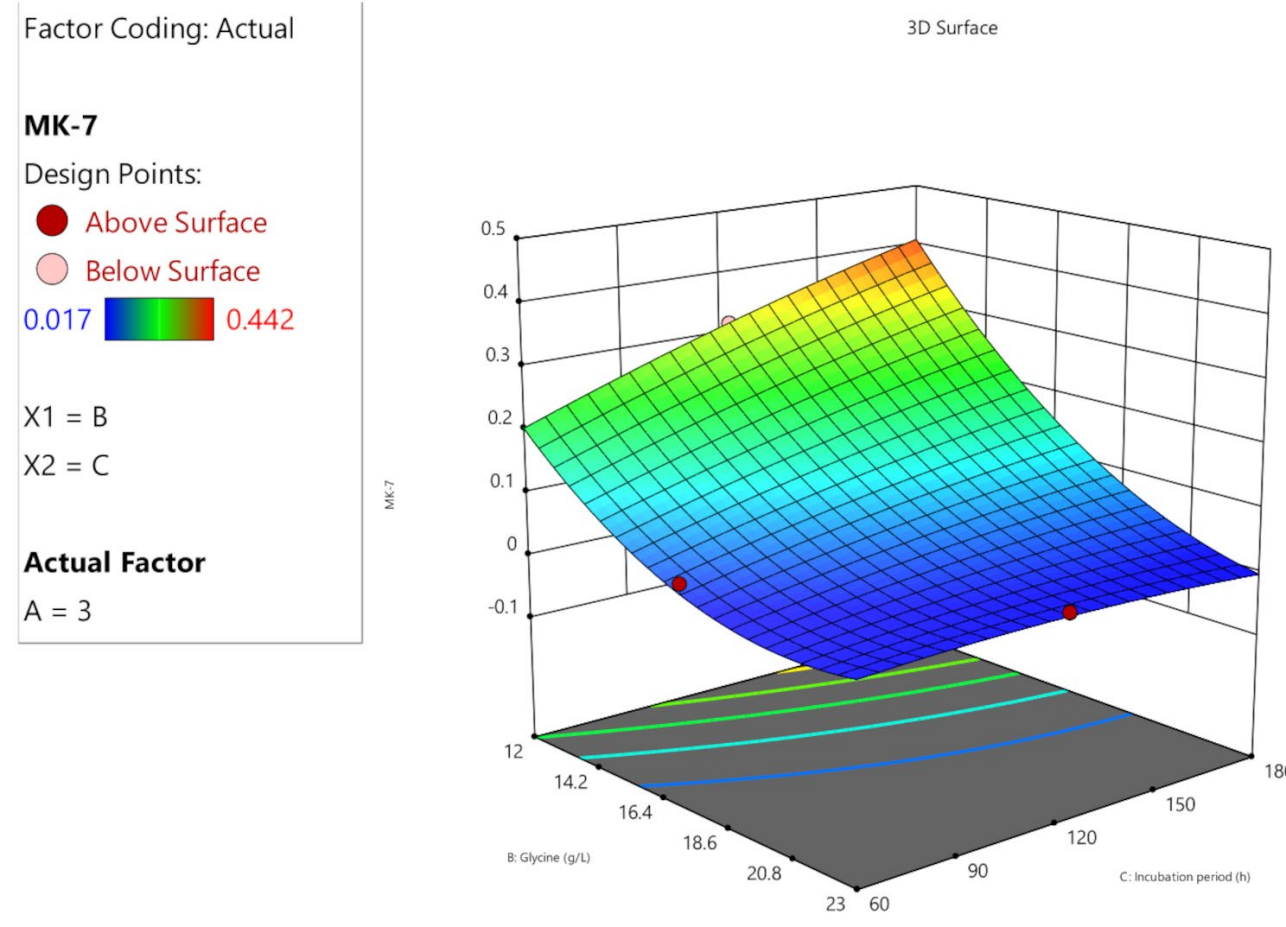
3-D response surface plot showing the interaction between nitrogen source and incubation time for the optimization of MK-7 production medium

## Discussion

The present study delves into synthesis and optimization of MK-7 in production media by employing *Bacillus subtilis* MM26, a potent strain obtained from fermented home-made wine. According to this study, the *Bacillus subtilis* MM26 inoculated in a medium containing yeast extract (0.5 g/mL), soy peptone (1.89 g/mL), glycerol (0.5 w/v mL), K2HPO4 (0.06 g/mL) generated MK-7 to a greater extent than the TSB, LB and NB medium. An MK-7 content of 0.123 mg/L was generated by lactic acid bacteria in soymilk medium [25]. The strain *Bacillus amyloliquefaciens* generated MK-7 of 11.3 ± 0.6 mg/L in the *cheonggukjang* fermentation medium [26]. Another study showed that *Bacillus cereus* KMV07 generated MK-7 of 16.74 ± 1.21 mg/L in an ideal fermentation medium [27]. According to Sato et al., *Bacillus subtilis* produced MK-7 of 60 mg/L in the fermentation medium [7]. Another research showed that *Bacillus amyloliquefaciens* Y-2 isolated from douche, exhibited MK-7 content of 11.8 ± 0.7 mg/L [27]. Previous studies showed that *Bacillus licheniformis* produced a content of MK-7 of 12.507 mg/kg [28]. The strain *Bacillus subtilis natto* yielded 36.366 mg/L in a fermentation medium containing 2% soy peptone, 2% tryptone, 1% glucose, 2% yeast extract and 0.1% CaCl2 [29] and the strain *Bacillus velezensis* ND yielded 43.8 ± 2.8 mg/L of MK-7 when raw soybean flour fermentation broth [30]. The strain *B. amyloliquefaciens* synthesized MK-7 of 73.57 ± 1.61 mg/L via protoplast fusion and atmospheric and room temperature plasma mutagenesis [31].

In this study, *Bacillus subtilis* MM26 yielded MK-7 of 67 ± 0.6 mg/L at day 5, which was quite high when compared to the MK-7 yield found in previous studies. The synthesis of MK-7 requires carbon sources because the mechanism to produce the quinone skeleton (1,4-naphthoquinone) and isoprene side chain depends on the fermentation medium [32]. According to another research, adding maltose to the medium caused *Bacillus cereus* to generate MK-7 of 18.53 ± 1.04 mg/L [33]. According to the majority of research, the effective source of carbon for increasing the production of MK-7 are glucose and glycerol [28, 34–37]. MK-7 synthesis rose to 71.8 mg/L when glycerol and sucrose were used in combination [22]. When soluble starch and sucrose was employed as carbon source, the strain *Bacillus subtilis* generated MK-7 of 7.3 ± 0.77 and 10.36 ± 0.61 [21]. Similarly, the growth and metabolism of microbes, as well as the development of proteins associated with cellular respiration activities rely on nitrogen sources in the fermentation medium [36, 38]. The strain *Bacillus subtilis* produced 16.35 ± 0.38 and 14.21 ± 1.01 of MK-7 when soy peptone and yeast extract was used as the nitrogen source [21]. In another study, combining soymeal and yeast extracts increased the content of Mk-7 and the growth of *B. amyloliquefaciens* subsp. strain FZB42, yielding 31.7 ± 2.5 mg/L and 25.7 ± 1.8 mg/L of MK-7 [27]. The MK-7 yield is significantly influenced by other factors including pH, temperature, inoculum size and incubation period [14]. The yield of MK-7 at 30 and 37 °C was found to be 0.319 and 0.3158 mg/L [39]. Higher content of MK-7 was noted at 35 °C with a pH of 6.58 [14]. According to another study, a maximum of MK-7 of 15.80 mg/L was produced at an ideal pH of 7.5 [22]. A content of 60 mg/L MK-7 was generated by the strain *Bacillus subtilis* MH-1 in a fermentation medium with a 7.3 pH at 45 °C [7]. *Bacillus cereus* KMV07 generated MK-7 of 15.80 ± 1.00 mg/L at pH 7.5 [33]. Another study showed that *Bacillus subtilis* produced a concentration of MK-7 of 18.45 ± 0.76 mg/L at 30 °C in a medium (pH 6.47) [40]. A study showed that *Bacillus subtilis* produced 23.54 ± 0.91 and 23.32 ± 0 of MK-7 at pH 7 and 7.5 [21]. The maximum MK-7 yield of 54 mg/L utilizing an inoculum size of 2 mL was also reported [22]. The maximum concentration detected was 46.98 ± 3.1 mg/kg dry weight of MK-7, with the lowest inoculation dose of 8.4 CFU [41]. Another study showed that a 3% inoculum size of culture (8.25 × 10^6^ CFU/mL) produced MK-7 of 20.20 ± 1.54 mg/L [33]. In this study, *Bacillus subtilis* MM26 yielded MK-7 of 65 ± 2.76 mg/L at 37 °C, 69 ± 0.57 mg/L at neutral pH 7 and 68 ± 0.57 mg/L when the inoculum was 2.5% (2 × 10⁶ CFU/mL). In this study, when lactose and glycine were used as the carbon and nitrogen source, the strain *Bacillus subtilis* MM26 produced 69 ± 1.20 mg/L and 69 ± 1.79 mg/L of MK-7. This yield was notably higher compared to values reported in previous studies mentioned. Also, the strain *Bacillus subtilis* MM26 produced 65 ± 2.76 mg/L at 37 °C of MK-7 and 69 ± 0.57 mg/L at neutral pH 7. Additionally, the strain produced 68 ± 0.57 mg/L of MK-7 when the inoculum was 2.5% (2 × 10⁶ CFU/mL). Overall, the MK-7 yield observed in this study represents approximately a 3.8-fold increase compared to previously reported values.

Using the OFAT method, five parameters that had significant influence on MK-7 synthesis were chosen for the preliminary investigation. It has been observed that the strain *Bacillus subtilis* MM26 was not able to produce MK-7 when the medium was supplemented with carbon sources including dextrose and maltose and peptone as nitrogen source. The strain produced 69 ± 1.20 mg/L and 69 ± 1.79 mg/L of MK-7 when lactose and glycine were added to the fermentation medium. This increase in the MK-7 yield led to an evaluation of the specific roles of each optimized variable, especially glycine and lactose. From the study it has been found that incubation time, carbon and nitrogen sources tend to have a vital role in the synthesis of MK-7 by *Bacillus subtilis* MM26 and were chosen for statistical optimization using RSM to maximize the MK-7 synthesis. The effect of glycine was demonstrated by comparing all the runs with 12th run. When glycine was increased to 17.5 g/L or 23 g/L as in the 17th run and 9th run, the MK-7 was decreased to 180 ± 3.17 and 80 ± 0.56 mg/L, respectively, whereas MK-7 yield was increased to 442 ± 2.08 mg/L at 12 g/L glycine in the 12th run. Similarly low or high concentration of lactose lead to poor MK-7 yield. These findings imply that MK-7 synthesis was ideally enhanced by optimal concentrations of glycine and lactose but greater amounts may cause metabolic disruption, suggesting a threshold effect. The study reported 6 g of lactose, 12 g of glycine and period of incubation of 180 h are the optimal variables for the maximum MK-7 production. It is also essential to consider strain’s metabolic flexibility in the MK-7 optimization. By combining *B. subtilis* MM26 with Box-Behnken Design statistical optimization, it is able to enhance MK-7 production and to demonstrate how crucial it is to modify fermentation conditions according to strain-specific responses.

Another study reported by Wu and Ahn [26], 3 variables were examined for the MK-7 optimization by *Bacillus subtilis* strain KCTC 12392BP and maltose, glycerol and tryptone were determined to be the most effective variables. The strain yielded a total of 71.95 ± 1.00 µg/ml of MK-7 at 9th day [26]. In order to determine the ideal fermentation media for the *Bacillus subtilis natto*, tryptone, yeast extract, CaCl_2_, soy peptone, and glucose were examined further in an optimization study utilizing a RSM and CCF design which produced MK-7 of 36.366 mg/L [29]. Another study investigated 4 different variables as a source of nitrogen and carbon for the optimized MK-7 synthesis and the yield was noted to be 62.32 0.34 mg/L [42]. Tween-80, glycerol, and soy peptone had a substantial impact on MK-7 synthesis (95.03 ± 1.01 mg/L) in *Bacillus amyloliquefaciens*, according to another RSM study [31]. Another research used the Plackett–Burman experimental design to screen for the MK-7 synthesis and the three variables were optimized using the Box–Behnken design. *Phaseolus vulgaris* generated MK-7 of 31.35 µg g^− 1^ in an optimized medium that contained 54.05 g kg^− 1^ of glycerol, 3.40 g kg^− 1^ of urea, and 15.00 g kg^− 1^ of soybean extract [43]. In an ideal medium consisting of 16.88 g L^− 1^ of yeast extract, 1.4 g L^− 1^ of l-glutamate, 130.95 g L^− 1^ of soybean meal, and 72.19 mL L^− 1^ of glycerol employing RSM, *Bacillus velezensis* generated a higher MK-7 content of 46.88 mg L^− 1^ [44]. The mathematical model of RSM demonstrated the maximum production of MK-7 of 63.05 mg/kg at 35℃ within 4 days with 10 µL/g of amylase and 1:1 wt of corn grits soy and protein granules [19]. Another study used central composite face design for the optimization and found that the addition of 0.32% (w/v) CaCl_2_ and 0.10% (w/v) urea to the medium resulted in the reduction of biofilm biomass and the maximum production of MK-7 of 17.98 mg/L [45]. The aspect of this study involved the application of RSM in increasing MK-7 production by *Bacillus subtilis* MM26 via optimization of lactose, glycine, and incubation time. Box-Behnken design has been used for analysis of interaction of the above factors. Results were modeled using a quadratic equation with corresponding high F-value of 27.76, *p*-value < 0.0001 and R² of 0.9727 indicating a strong fit. Glycine was the variable that most significantly affected MK-7 synthesis, followed by lactose and incubation time. The predicted and actual responses were closely aligned, indicating a high accuracy. Response surface plots revealed the combined effects of the variables, validating their critical role in maximizing MK-7 yield (442 ± 2.08 mg/L). The MK-7 yield was increased approximately 6.4-fold, from an initial production of 69 mg/L to 442 mg/L after RSM optimization. Compared with the previously mentioned research studies, the strain *Bacillus subtilis* MM26 showed a significant improvement in MK-7 production, with a 4.65-fold increase. The HPLC chromatogram exhibited a single peak with a retention time of 4.9 min which was compared to the standard MK-7 [18]. Significant peaks were seen in the sample’s FTIR spectra at 1660 cm − 1 and 1536 cm⁻¹, which correspond to the carbonyl groups on the naphthoquinone ring and amide II, respectively. The observation aligns with earlier research and validates the existence of MK-7 [20]. Although earlier research provided information regarding different fermentation methods, this research extends further by combining optimization strategies with a deeper understanding of the underlying process, resulting in a substantial increase in MK-7 synthesis. This highlights the critical role of strain-specific media composition and statistical optimization in improving MK-7 production.

## Conclusion

Menaquinone-7 is a vital nutrient with several health benefits. It’s superior bioavailability along with its role in regulating calcium and diminishing the risks of cardiovascular diseases as well as other disorders, underscores the importance of MK-7 in human diet. According to the present research *Bacillus subtilis* MM26, isolated from fermented home-made wine, has the potential to be an efficient MK-7 producer. The MK-7 yield was significantly increased to 442 ± 2.08 mg/L, by combining OFAT with RSM. Future research will be focused to enhance MK-7 yield by metabolic and genetic engineering of *Bacillus subtilis* MM26 followed by cost-effective downstream processing strategies to make MK-7 production more economical.

## Data Availability

No datasets were generated or analysed during the current study.

## Abbreviations

MK-7: Menaquinone − 7
HPLC: High performance liquid chromatography
FTIR: Fourier Transform Infrared
OFAT: One factor at a time
RSM: Response surface methodology
TSB: Tryptic soy broth
LB: Luria Bertani broth
NB: Nutrient broth
